# Tracking Public Interest in Rare Diseases and Eosinophilic Disorders in Germany: Web Search Analysis

**DOI:** 10.2196/69040

**Published:** 2025-05-26

**Authors:** Michael Hindelang, Sebastian Sitaru, Alexander Zink

**Affiliations:** 1 Department of Dermatology and Allergy, TUM School of Medicine and Health, Technical University of Munich, Ismaningerstraße 22, Munich, 81675, Germany, 49 894140 ext 4022; 2 Pettenkofer School of Public Health, Munich, Germany; 3 Institute for Medical Information Processing, Biometry, and Epidemiology (IBE), Ludwig-Maximilians-Universität München, Munich, Germany

**Keywords:** hypereosinophilia, eosinophilia, public health informatics, web search analysis, rare diseases

## Abstract

**Background:**

Eosinophilia and hypereosinophilic syndrome (HES) are rare disorders grouped under the term hypereosinophilic disorders. They are diagnosed based on an increased number of eosinophils. They can also cause serious symptoms, including skin, lung, and gastrointestinal problems. These disorders are very rarely recognized due to their rarity and misdiagnosis.

**Objective:**

This study analyzes public interest in hypereosinophilic disorders using data on internet search volume in Germany between 2020 and 2023. Objectives include identifying frequently searched terms, evaluating temporal trends, analyzing seasonal patterns, evaluating geographic differences in search behavior, and identifying unmet information needs and frequently searched risk factors.

**Methods:**

A retrospective analysis using Google Ads Keyword Planner gathered monthly search volume data for 12 German terms related to hypereosinophilic disorders. These terms were selected based on their medical relevance and common usage identified from medical literature. Data were analyzed descriptively, with trends, seasonal variations, and geographical distributions examined. Chi-square tests and correlation analysis assessed statistical significance.

**Results:**

A total of 178 keywords were identified, resulting in a search volume of 1,745,540 queries. The top keyword was “eosophile,” a misspelling, followed by “eosinophilia” and “HES.” The main categories included “Eosinophilia,” “Eosinophils,” and “Churg-Strauss syndrome.” Temporal analysis showed seasonal growth in search volumes, peaking in January 2023, with higher interest during winter. Geographical analysis showed regional variations.

**Conclusions:**

This research shows a growing public interest in eosinophilic diseases, reflected by a steadily increasing search volume over time. This is particularly evident in searches for basic definitions and diagnostic criteria, such as “eosinophils” or “symptoms of eosinophilic diseases.” This increase in search volume, which peaked in January 2023, indicates an increased interest in accurate and readily available information for rare conditions.

## Introduction

### Background

Hypereosinophilic diseases are defined as the presence of persistently elevated eosinophil counts that can cause tissue damage and inflammation of various organs, including the skin, lungs, or gastrointestinal tract [1,2]. These diseases cause symptoms such as severe itching, breathing problems, and gastrointestinal complaints [1,3-5]. Some severe forms of these diseases are characterized by hypereosinophilic syndrome (HES), in which there is tissue infiltration of eosinophils above accepted thresholds for eosinophilia, which can lead to abnormal damage to organ systems [6-9].

Hypereosinophilic disorders can have a significant impact on the individual, but they are often unrecognized and misdiagnosed, mainly due to their rarity and the heterogeneity of clinical presentation [10-13]. Diagnosis is typically based on the exclusion of other causes of eosinophilia (eg, allergic diseases or parasitic infections) and histopathological and immunohistochemical studies, permitting classification in the following subtypes: myeloproliferative (M-HES), lymphocytic (L-HES), idiopathic (I-HES), and chronic eosinophilic leukemia not otherwise specified (CEL-NOS). [1,10]. Treatment is based on subtype; corticosteroids plus systemic immunosuppression may be used, as well as cytotoxic agents (hydroxyurea and methotrexate), tyrosine kinase inhibitors (imatinib for M-HES), or targeted biologics (mepolizumab) [4]. Prognosis for HES varies; mortality rates can be as low as 7% to 10% for some subtypes, while mortality rates for CEL-NOS can be as high as 33% over a period of 19‐90 months [4].

It would be helpful to understand how the public navigates information about hypereosinophilic disorders to identify how this process could be altered to lead to successful diagnosis and treatment [4,14]. Digital technologies have developed to the extent that there is widespread access to online health information [15]. Patients are also more likely to turn to the internet to research their health-related questions, starting with a search for their specific symptoms and continuing with possible underlying causes and treatment plans [16-21]. Therefore, this shift from patients becoming passive recipients of medical advice to active participants in health care has seen a steep increase.

The patient journey—the patient experience from symptom awareness to treatment itself—has become more fluid and individualized [15]. This process may involve many phases, such as seeking signs of symptoms, possible diagnoses, or treatment options. These phases are dependent on individual, emotional, and contextual factors and lead to a series of nonlinear pathways through the health care system. These pathways are particularly convoluted in hypereosinophilic disorders [22] due to changing patient presentations, providers, diagnostic challenges, and lack of public and clinical awareness.

The Google Ads Keyword Planner is a valuable tool that allows users to analyze public interest and engagement with health topics using search volume data [23,24]. This approach allows researchers to monitor real-time data on public interest, identify trends in information tracking, and analyze spatial and temporal variations in interest [25-27]. In contrast, Google Trends provides a broader view of relative interest over time, offering insights into how frequently terms are searched relative to all searches made on Google. While Google Trends normalizes search volume data and provides general trends, it lacks the detailed search volume metrics that Google Ads Keyword Planner offers. Unlike social media, where shared information may be curated and filtered [28], search queries provide a more direct and unfiltered view of individuals’ health concerns and information needs. This perspective is particularly relevant for rare and underrecognized conditions such as hypereosinophilic disorders, where traditional sources of public health data may be limited.

### Objective

Given the rarity and diagnostic challenges of hypereosinophilic disorders, understanding how the public seeks information about these conditions can provide valuable insights into awareness gaps and unmet informational needs. Therefore, the primary aim of this study is to analyze public interest and information-seeking behavior related to eosinophilic disorders in Germany, using Google search data from 2020 to 2023. By examining trends, seasonal patterns, and geographical variations in search volumes, this study seeks to identify key areas of concern and opportunities to enhance public education on these underrecognized conditions.

## Methods

### Study Design and Data Collection

In this retrospective analysis, the Google Ads Keyword Planner was used to gather monthly search volume data. Although initially designed for marketing campaigns, this tool effectively provides monthly web search volume data (ie, monthly number of web searches) for research purposes [27,29-31]. To determine the search volume in a specific area, relevant search terms are entered into the planner. The language and geographical settings can then be configured, and the most relevant keywords and phrases for the topic entered.

### Search Terms and Keyword Identification

**Figure 1.**
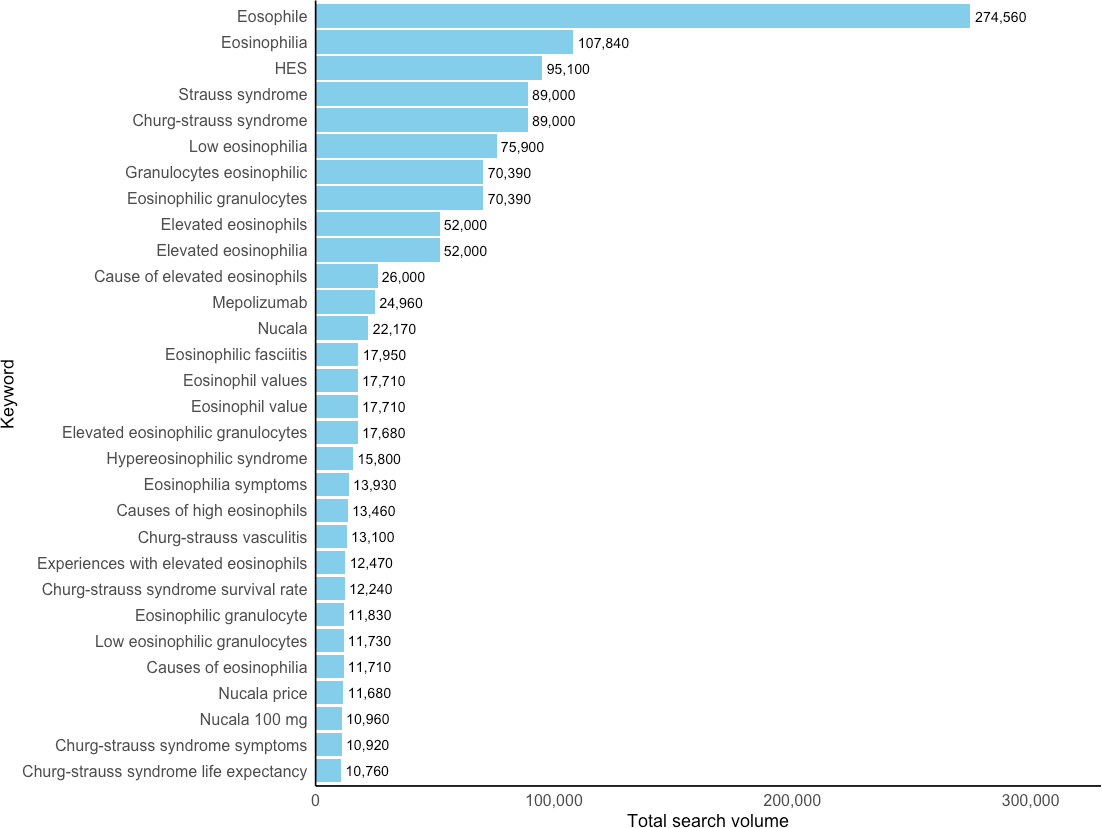
Total search volume for the top 30 keywords (2020‐2023). HES: hypereosinophilic syndrome.

For this study, 12 German search terms related to hypereosinophilia and associated conditions were entered (Figure 1). The goal was to obtain related keywords and phrases and their monthly search volume in Germany between January 2020 and December 2023. The search terms were “Hypereosinophilia,” “Hypereosinophilic Syndrome,” “Hes,” “Eosinophilia,” “Blood Eosinophilia,” “Reactive Eosinophilia,” “Tissue Eosinophilia,” “Eosinophilic Granulocytes,” “Eosinophilic Syndrome,” “FIP1L1,” “Mepolizumab,” and “Nucala.”

The 178 keywords were reviewed for relevance to hypereosinophilia and grouped inductively into categories based on their association with the disease, clinical subtypes, treatments, diagnostic markers, and related conditions: “Eosinophilia,” “Eosinophils,” “Churg Strauss Syndrome,” “HES,” “Eosinophilic Granulocytes,” “Nucala,” “Mepolizumab,” “Eosinophilic Fasciitis,” “Eosinopenia,” “EGPA (Eosinophilic Granulomatosis with Polyangiitis),” “Eosinophilic Granulomatosis,” “Blood Eosinophilia,” and “FIP1L1.”

Categories for recurring topics were further subdivided into subcategories (eg, diagnostic information). For each keyword, only one subcategory was assigned. The data were analyzed descriptively.

### Geographical Scope

The search volume in all of Germany was examined. Search data for all 16 German federal states and cities were analyzed. Cites were selected based on their population and geographic location in order to obtain a representative overview of all of Germany. For a more in-depth view, cities that are of particular interest due to their unique demographics or health-related infrastructure were also included.

The predefined list of cities included in the study comprised cities that are well distributed across the country and include both large and small cities: Bad Bramstedt, Berlin, Bremen, Cologne, Dortmund, Dresden, Erfurt, Frankfurt, Freiburg, Giessen, Hamburg, Hanover, Heidelberg, Jena, Kassel, Kiel, Kirchheim Teck, Leipzig, Magdeburg, Mainz, Mannheim, Munich, Nuremberg, Regensburg, Rostock, and Stuttgart.

### Statistical Methods

To summarize and visualize the search volume data, we used descriptive statistics. Means and measures of dispersion (SD and IQR) were calculated for the monthly search volumes in different categories and subcategories. Frequencies and percentages were used to describe the distribution of searches among the identified categories. We applied a time series decomposition to monthly search volume data since January 2020, using the seasonal and trend decomposition using Loess to extract seasonal, trend, and remainder components. This allowed us to quantify seasonal patterns with CIs and to measure the variability in the data.

### Temporal and Geographical Analysis

Trends during the study reporting period (January 2020 to December 2023) were assessed using a time series analysis. We applied seasonal decompositions to locate and quantify seasonal fluctuations in search volume (seasonal decomposition of time series). Search queries were processed for each federal state and city, and the search queries per 100,000 inhabitants were calculated to analyze the geographical distribution of the search volume. This allowed us to identify areas with increased or decreased search activity related to eosinophilic disorders.

### Statistical Testing

Chi-square tests were conducted to assess the significance of differences in search volume between categories and regions. This test was useful for determining whether the distribution of searches across categories was statistically different from what would be expected by chance.

### Correlation Analysis

Prior to statistical analysis, we tested the normality of the search volume data using the Shapiro-Wilk test. The results indicated a normal distribution (*P*=.89). A chi-square test was conducted to examine the associations between search volumes and different regions. This analytical strategy helped to explore potential demographic or health-related factors associated with higher search activity. In this map, each tile represents a search term category, and red areas highlight regions with high relative search volume, resulting in an easy-to-understand heat map visualization that makes it simple to see how interest is distributed across regions in the categories. The intensity of the displayed color reflects the number of searches per 100,000 people on average, with light colors indicating minimal search interest and dark colors indicating higher search interest.

### Identification of Significant Rises

We calculated *z* scores for each time point to detect significant increases in search volume. The *z* score was computed by subtracting the mean search volume and dividing by the SD. Points with *z* scores greater than 2 were marked as significant rises, indicating substantial deviations from the average trend.

### Validation of Methodology

To ensure the validity of the methodology, the search volumes from the Google Ads Keyword Planner were compared with data from Brandwatch (Giles Palmer) [32]. Brandwatch analyzes mentions on various platforms such as Facebook Public, forums, internet-based news, and X (formerly Twitter). This comparison was used to verify whether the identified peaks and trends in search volume can also be found on other platforms and whether similar patterns exist across multiple sources. This ensured that the observed trends are consistent and not distorted by the commercial orientation of Google Ads.

### Software and Tools

All statistical analyses were performed using R (version 4.1.2; R Core Team). Spatial data analysis used *rnaturalearth* packages, and visualizations were created with *ggplot2* [33-40].

### Ethical Considerations

As the study was based on publicly accessible Google search terms, there was no requirement for institutional review board approval, and informed consent was not applicable.

## Results

### Overview of Search Volume

Overall, 178 keywords related to hypereosinophilia were identified, resulting in a search volume of 1,745,540 queries from January 2020 to December 2023.

The analysis of search volumes for keywords related to eosinophilic disorders revealed several key insights. Interestingly, the top keyword was “eosophile” with 274,560 searches, despite being a misspelling and lacking medical meaning (Figure 1). This indicates a potential gap in public understanding or a common typographical error. Following this, the correct medical term “eosinophilia” had 107,840 searches, and “HES” had 95,100 searches. Other top keywords with the highest search volumes included “Churg-Strauss syndrome” and “low eosinophilia” (Table 1).

**Table 1.** Top 30 unique keywords for each main category and subcategory.

Main category	Subcategory	Frequency of unique keywords, n
Eosinophilia	Diagnosis	18
Eosinophil granulocytes	Diagnosis	15
Eosinophils	Diagnosis	13
Eosinophilia	Causes/associated diseases	11
HES^a^	General information	11
Nucala	General information	11
Mepolizumab	General information	10
Eosinophilia	Symptoms	7
Churg-Strauss syndrome	Treatment	5
Churg-Strauss syndrome	General information	5
Eosinophilic fasciitis	General information	5
Eosinophilia	General information	5
Churg-Strauss syndrome	Causes/associated diseases	4
Eosinophilia	In animals	4
Churg-Strauss syndrome	Diagnosis	3
Churg-Strauss syndrome	Symptoms	3
Eosinopenia	General information	3
Nucala	Dosage	3
Nucala	Costs	3
Blood eosinophilia	General information	2
Churg-Strauss syndrome	Localization	2
Churg-Strauss syndrome	Survival	2
EGPA^b^	Treatment	2
Eosinopenia	Diagnosis	2
Eosinophil granulocytes	Causes/associated diseases	2
Eosinophilia	Treatment	2
Eosinophils	Causes/associated diseases	2
HES	Treatment	2
HES	Symptoms	2

a HES: hypereosinophilic syndrome.

b EGPA: eosinophilic granulomatosis with polyangiitis.

### Categorization of Search Terms

**Figure 2.**
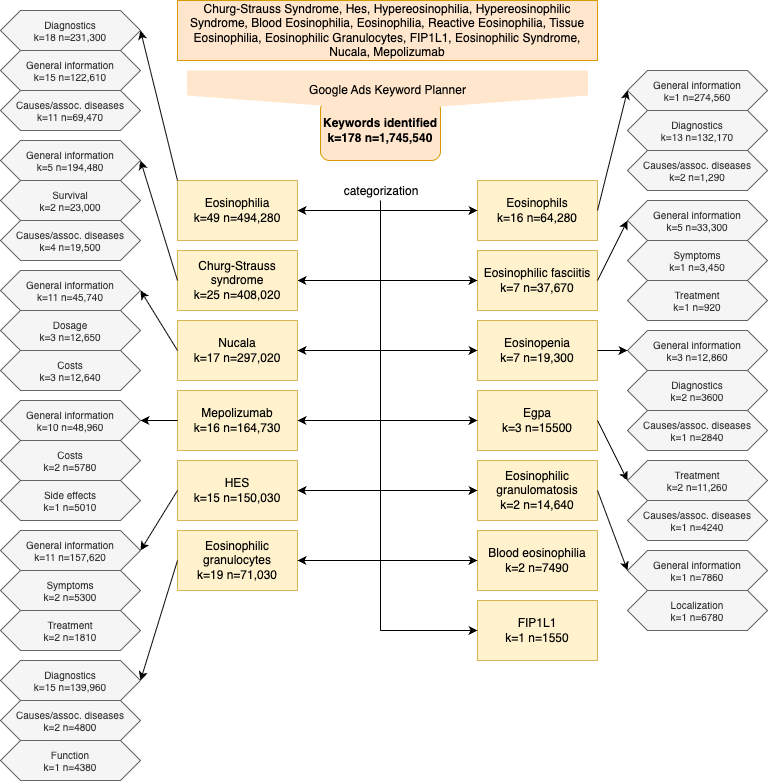
Flowchart of data generation and content categorization. This flowchart illustrates the process used to gather and categorize the keywords related to eosinophilic disorders. First, relevant search terms were identified based on their medical significance and prevalence in the literature. Next, data were collected from Google Ads Keyword Planner, which provided monthly search volume information for each term. The identified keywords were then grouped into broad categories such as “Eosinophilia,” “HES,” “Churg-Strauss Syndrome,” and others based on their relevance. Within these categories, further subcategories were created, focusing on specific topics such as diagnosis, symptoms, treatment, and related diseases. For clarity, only the top three subcategories are listed when more than three subcategories exist. HES–related web searches in Germany from 2020 to 2023 were analysed. If no subcategory was created, the group was too small or only general information was searched for. assoc.: associated; EGPA: eosinophilic granulomatosis with polyangiitis; FIP1L1: gene involved in hypereosinophilia; HES: hypereosinophilic syndrome; k: number of keywords; n: number of searches.

These keywords were assigned to the following categories (Figure 2). In terms of main categories, “Eosinophilia” topped the list with a total of 494,280 searches, accounting for 28.32% of the total search volume. This was followed by “Eosinophils” with 408,020 searches (23.38%) and “Churg-Strauss syndrome” with 297,020 searches (17.02%). These categories highlight the primary areas of interest among the public.

Within these main categories, the top subcategories provided further insight into specific areas of interest (Figure 2). For “Eosinophilia,” the most searched subcategory was “diagnosis” with 231,300 searches (13.25% of the total search volume), followed by “general information” with 122,610 searches (7.02%), and “causes/associated diseases” with 69,470 searches (3.98%). For “Eosinophils,” the leading subcategories were “general information” (274,560 searches, 15.73%), “diagnosis” (132,170 searches, 7.57%), and “causes/associated diseases” (1290 searches, 0.07%). In the “Churg-Strauss syndrome” category, “general information” led with 194,480 searches (11.14%), followed by “survival” with 23,000 searches (1.32%) and “causes/associated diseases” with 19,500 searches (1.12%).

### Temporal Trends in Search Volume

**Figure 3.**
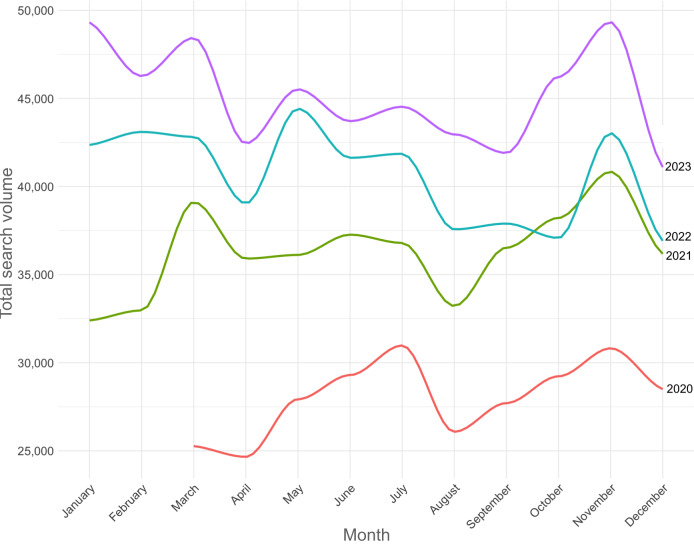
Total search volume by month over 4 years.

From March 2020 to December 2023, eosinophilic disorders exhibited significant growth, peaking in January 2023 at 49,320 queries (Figure 3). Seasonal patterns reveal higher interest during winter months and slight declines in summer, with a consistent yearly increase in overall search volumes. This trend highlights rising public awareness and interest in eosinophilic disorders over time (see also Figure S3 in Multimedia Appendix 1) .

The decomposition of the search volume time series revealed key patterns (Figures S5 and S6 in Multimedia Appendix 1), with Figure S5 in Multimedia Appendix 1 outlining the seasonal and trend decomposition using Loess breakdown into seasonal, trend, and remainder components, and Figure S6 in Multimedia Appendix 1 confirming the seasonal estimates’ reliability through diagnostic plots and CIs. The seasonal component showed periodic fluctuations, with notable peaks and troughs, such as a peak in March 2020 (2889) and a low in December 2020 (–4165). This indicates regular cyclical variations in search volume. The trend component exhibited a consistent upward trajectory, increasing from 24,925 in March 2020 to 46,654 by December 2023, suggesting sustained growth in search interest. The remainder component displayed random fluctuations, reflecting irregular variations not explained by the seasonal or trend components. CIs for the seasonal component showed variability, particularly in March 2020, with intervals ranging from –1687 to 7467. Over time, these intervals became narrower, indicating more precise seasonal estimates.

### Geographic Distribution

**Figure 4.**
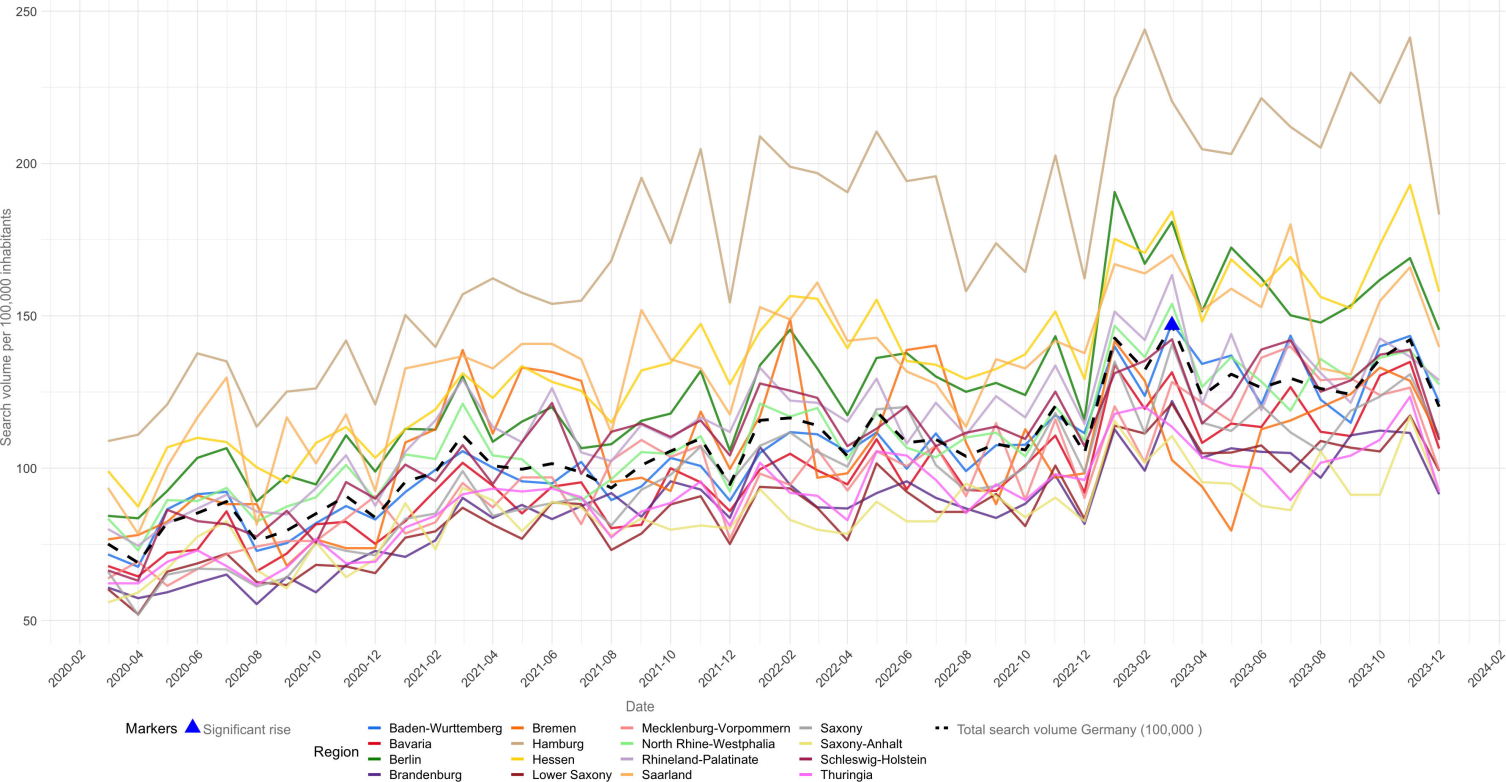
Total search volume over time per 100,000 inhabitants for Germany and by state.

From March 2020 to December 2023, the total search volume per 100,000 inhabitants in Germany showed notable variations (Figure 4 and Figure S1 in Multimedia Appendix 1). Nationally, the peak search volume was 147 per 100,000 inhabitants in March 2023, and the lowest was 69 in April 2020. At the state level, Hamburg recorded the highest peak of 244 searches per 100,000 inhabitants in February 2023. In contrast, Saxony-Anhalt’s peak was 117 in November 2023. Seasonal patterns were observed, with increased search volumes across most regions at the start and end of the year. The lowest volumes generally occurred in April 2020 across various states, reflecting consistent national trends.

**Figure 5.**
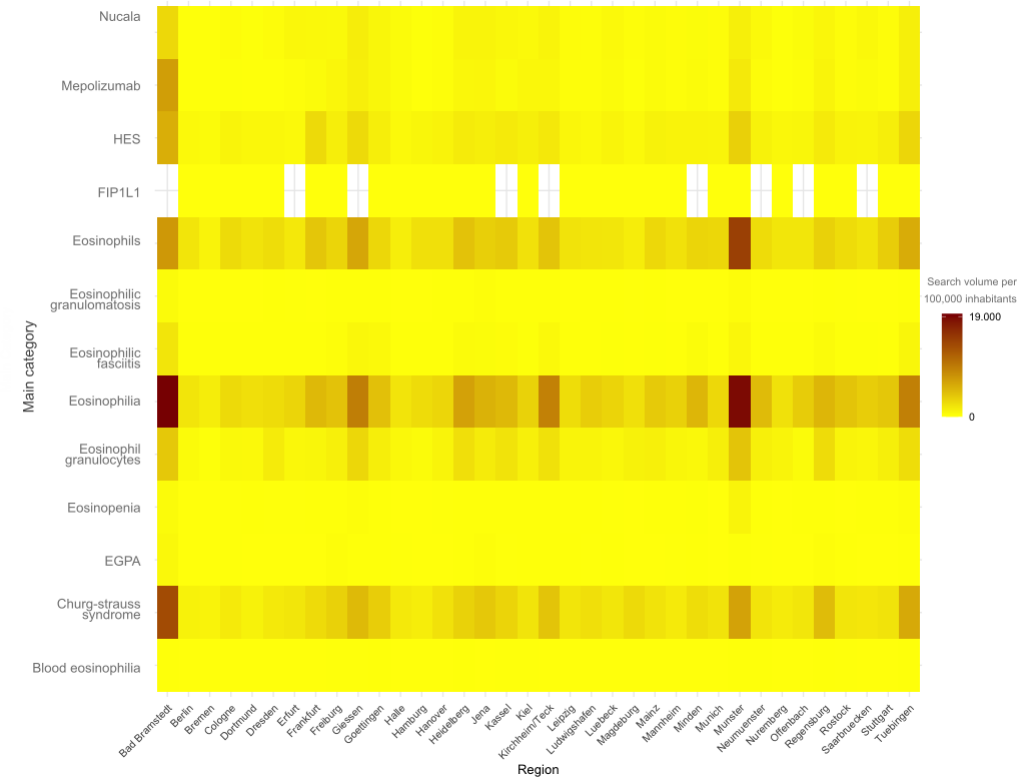
Search volume per 100,000 inhabitants for key categories across regions in Germany from 2020 to 2023. EGPA: eosinophilic granulomatosis with polyangiitis; FIP1L1: gene involved in hypereosinophilia; HES: hypereosinophilic syndrome.

The heatmap (Figure 5 and Figure S4 in Multimedia Appendix 1) ) illustrates the search volumes per 100,000 inhabitants across the different German cities. “Eosinophilia” exhibits the highest search volume in Bad Bramstedt with 19,523, while Bremen shows the lowest with 1513. Similarly, for “Churg-Strauss syndrome,” Bad Bramstedt has the highest value at 13,562, compared with Bremen’s 1043. For medications such as “Mepolizumab” and “Nucala,” the highest values are also in Bad Bramstedt (7004 and 2832, respectively), with Bremen having the lowest (207 and 218, respectively). Other cities such as Munster and Tuebingen also display high search volumes, particularly for “eosinophils” (14,529 in Munster) and “Churg-Strauss syndrome” (6154 in Tuebingen).

**Figure 6.**
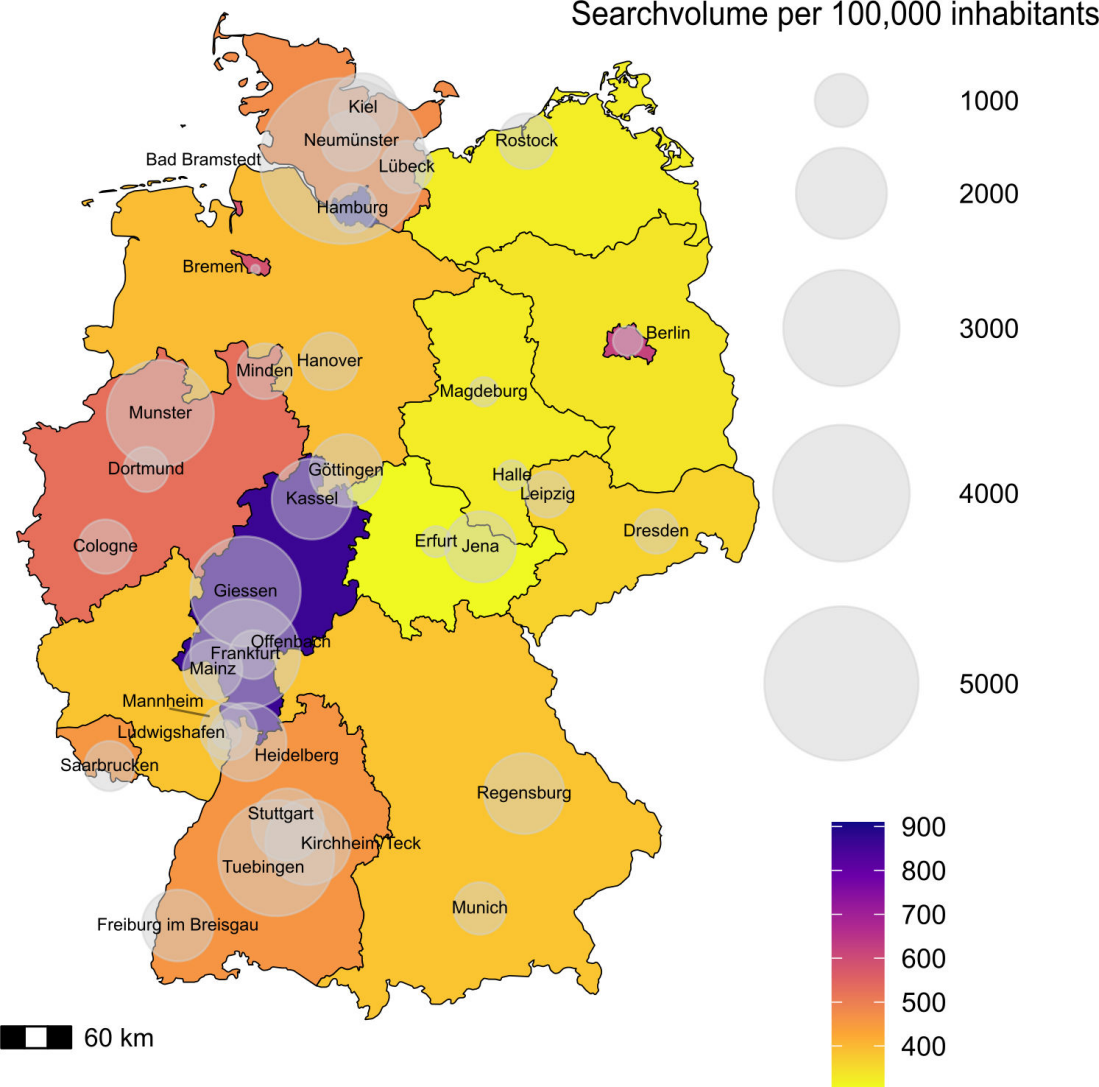
Search volume per 100,000 inhabitants for the “HES” category by region. HES: hypereosinophilic syndrome.

The analysis of the geographical distribution of the total 4-year search volume for the category “HES” per 100,000 inhabitants only shows remarkable differences across different regions and cities (Figure 6). Hamburg has the highest search volume at 854, followed by Hessen with 805. Thuringia, Saxony-Anhalt, and Mecklenburg-Vorpommern have the lowest volumes at 304, 318, and 327 per 100,000 inhabitants, respectively. Among cities, Bad Bramstedt has the highest search volume at 5738 per 100,000 inhabitants. Tübingen, Giessen, and Frankfurt follow with 2989, 2736, and 2719 respectively. Münster reports 2600 searches, Kirchheim Teck reports 1825, Kassel reports 1666, and Regensburg reports 1665. Heidelberg, Göttingen, Stuttgart, and Freiburg im Breisgau show volumes of 1598, 1441, 1433, and 1413, respectively. The lowest search volumes are in Erfurt, Halle, Berlin, and Magdeburg with 690, 683, 680, and 676, respectively, and Bremen has 587 searches per 100,000 inhabitants.

### Validation of Search Volume Trends With External Data Sources

To validate the findings from Google Ads Keyword Planner, we compared them with data from a platform [32] that analyzes mentions across social media, forums, internet-based news, and X (formerly Twitter) (Figure S2 in Multimedia Appendix 1). The data revealed notable peaks in January 2023, particularly in internet-based news, and a peak in October 2022 for forum mentions. These trends aligned with the spikes observed in the Google Ads search volume data, supporting the hypothesis that the increases in search activity are reflected in broader public discussions, indicating genuine rises in interest about hypereosinophilic disorders.”

### Correlation Analysis

The chi-square test result was highly significant (*P*<.001), indicating a strong association between the categories of search terms and the regions after normalization per 100,000 inhabitants. This suggests that the frequency and type of searches for HES–related information are significantly influenced by regional factors (Table S1 in Multimedia Appendix 1).

### Identification of Significant Rises

Significant rises in search volume are marked with blue triangles in Figure 4. These were detected using *z* scores, indicating periods where the search volume increased substantially beyond typical variations. Notably, in March 2023, there was a significant rise in search volume, reaching 147 per 100,000 inhabitants, well above the average with a *z* score of 2.08. This points to a notable event or increased interest during this period, potentially indicating a surge in public concern or awareness about the topics searched.

## Discussion

### Principal Findings

From March 2020 to December 2023, a total of 1,745,540 search queries on eosinophilic diseases were documented, suggesting a high level of public interest in eosinophilic diseases in Germany. The increasing number of searches conducted, particularly during the winter months, may indicate a growing public interest in rare diseases, possibly due to external influences (eg, seasonal worsening of symptoms and increased media coverage) or displacement effects on search engine suggestion lists. It is striking how often people enter search terms incorrectly, such as “eosophile,” which accounted for 274,560 search queries, suggesting gaps in the public’s understanding of medical terminology. The frequent misspelling of terms indicates a barrier to finding correct medical information. These spelling mistakes show that there are gaps in the public’s knowledge of medical terms, which also points to the need for digital literacy campaigns. Raising awareness and educating the public about the correct terminology can help improve the search for information and support self-education, especially with regard to rare diseases.

Regional differences in search volume suggest that cities like Hamburg, Bad Bramstedt, and Freiburg show higher engagement, likely due to the presence of specialized health centers and greater access to medical information. In comparison, Saxony-Anhalt and Bremen show a lower search volume because there are fewer specialists in these regions and consequently less awareness of the disease. These rates may vary depending on disease prevalence and regional health campaigns, as indicated by the peak in January 2023. Future studies should investigate how access to health care, disease prevalence, and regional campaigns may impact public interest in rare diseases. Interestingly, the most common subcategory for searches related to the broader topic of eosinophilia was diagnosis, suggesting a desire among the public to learn more about the identification and symptoms of eosinophilic disorders. This suggests that those searching for such terms may not yet have been diagnosed and are looking for possible causes of their symptoms. The spike to 49,320 searches in January 2023 may be the result of public health campaigns or media coverage and illustrates how external events influence public health information behavior.

Insights into user preferences highlight some important issues related to eosinophilic diseases, particularly in the area of diagnosis. Conversely, public health efforts should focus on simplified and accessible materials for patients in the early stages, containing credible knowledge about symptoms and evaluations to support efficient and correct diagnosis. Searching and geographically examining keywords can enable targeted public health campaigns in unconscious areas, which can shorten the diagnostic delay and lead to earlier detection and better outcomes. Seasonal peaks in search volume, particularly during the winter months, may reflect worsening of respiratory symptoms due to the colder weather [41]. Due to the exacerbation of eosinophilic diseases, including HES, during this period, people feel the need for more information [41]. The peaks can also be explained by increased public health campaigns or media attention, as respiratory problems tend to receive more attention during the flu season [42].

Regions with lower engagement, such as Saxony-Anhalt and Bremen, may benefit from targeted outreach strategies to increase awareness and education about eosinophilic disorders. Health campaigns in these areas could focus on improving access to information, addressing gaps in public knowledge, and raising awareness about available specialized care centers [43-46]. Additionally, digital platforms, including social media and search engines, can be leveraged to disseminate accurate and timely information, directly reaching individuals in these regions who may not have access to traditional sources of health education. Such approaches could help reduce diagnostic delays by guiding individuals to seek appropriate care sooner and more effectively [43].

To validate the findings, data from social media and internet-based news were examined. The trends, especially the peaks in January 2023 and October 2022, align with search volume spikes. However, social media data may reflect specific user groups rather than the broader population, and news data can be influenced by external factors such as public health campaigns. While these sources provide useful insights, they should be interpreted cautiously as they may not fully capture the overall public interest in eosinophilic disorders.

### Comparison With Literature

The findings of our study are consistent with previous research on rare diseases, which highlights the significant challenges faced by patients in obtaining a timely and accurate diagnosis. Hypereosinophilic disorders present with complex clinical features that can lead to diagnostic delays and misdiagnoses, similar to other rare conditions. The 2013 report on the impact of rare diseases shows that patients with rare diseases often have to undergo long diagnostic pathways. In the United States, it takes an average of 7.6 years, and in the United Kingdom, 5.6 years before a correct diagnosis is made [44]. During this period, patients typically consult up to 8 physicians and receive 2-3 misdiagnoses, with 82% of social media comments on HES reporting diagnostic delays, highlighting the complexity of diagnosing rare eosinophil-driven disorders [45].

In our study, the diagnosis-related subcategory was the most frequently searched, reflecting the public’s struggle to find accurate information and the challenges associated with diagnosing hypereosinophilic disorders [46,47]. This pattern of information-seeking behavior suggests a significant unmet need for awareness and educational resources, both for the public and health care professionals [48].

This finding aligns with previous research on atopic dermatitis and pollen allergies, which also indicated that online search data could reveal significant gaps in public knowledge and help identify areas requiring targeted health education [26,29,49-51].

### Strengths and Limitations

A key strength of this study is its use of Google Ads Keyword Planner, providing real-time search data to assess public interest in eosinophilic disorders. This approach demonstrates the potential of infodemiology for rare disease research, offering insights into awareness, informational needs, and regional variations, which traditional data sources often lack. Compared with studies such as Pauer et al [52], which used search queries for rare disease epidemiology, our study focuses specifically on eosinophilic disorders, adding nuance to public engagement analysis [53,54]. Additionally, unlike Pauer et al [52] and Tozzi et al [55], which examined information quality and user demographics, we provide real-time trends in public interest, enhancing the understanding of disease awareness.

Several limitations should be considered when interpreting these findings. Google Ads Keyword Planner, while effective for data collection, is primarily a marketing tool and may introduce biases or limitations in data accuracy for research purposes [56,57]. The high search volume for misspelled terms such as “eosophile” may have skewed the results and points to gaps in public education that were not fully explored in this study. Future research could investigate the reasons behind common misspellings and their impact on information retrieval and understanding. Furthermore, the generalizability of the results may be limited by demographic and access biases. Younger people are generally more tech-savvy and therefore more likely than older cohorts to search for health information on Google, skewing the search data toward their demographic group. This study did not account for differences in internet use by age, socioeconomic status, and region, and the results may not generalize to different segments of the population [58]. The final limitation concerns the temporal relevance of the data, which extend to December 2023 but may not account for newer trends or developments, such as newer treatment options or educational campaigns, that could influence interest and search behavior outside the time frame of this study.

### Conclusion

The findings of this study provide valuable insights into public interest and information-seeking behaviors related to hypereosinophilic disorders in Germany. The data suggest that there is a growing awareness and concern about these rare conditions, as evidenced by the increasing search volumes over time. The pronounced regional differences highlight the need for localized health education and resource allocation to address potential disparities in awareness and access to information.

Our results emphasize the urgent need for faster, more accurate diagnostic pathways and enhanced awareness among health care professionals to improve the management and outcomes for patients with rare diseases such as HES and EGPA. The study underscores the potential of using Google search trends as a tool for public health surveillance, particularly for rare and underrecognized conditions such as hypereosinophilic disorders. Future research should aim to integrate demographic data and explore the impact of public health campaigns and health care access on search behaviors. Additionally, efforts should be made to improve public understanding of these disorders through accurate and accessible information, potentially leveraging the very platforms where information-seeking is occurring. Addressing these knowledge gaps could lead to better patient outcomes through earlier diagnosis and more informed health decision-making.

## Supplementary material

10.2196/69040 Multimedia Appendix 1 A correlation matrix of search volume data for various keywords related to eosinophilic disorders across 27 cities in Germany. This matrix highlights the relationships between different terms such as "eosinophilia," "Churg-Strauss syndrome," "HES," and related treatments and conditions. The data reflect regional variations with notable differences in search volume across cities. These correlations provide insights into public interest in specific aspects of eosinophilic disorders and help identify patterns in search behaviors linked to geographic location.

## Abbreviations

CEL-NOS: chronic eosinophilic leukemia-not otherwise specified
EGPA: eosinophilic granulomatosis with polyangiitis
HES: hypereosinophilic syndrome
I-HES: idiopathic hypereosinophilic syndrome
L-HES: lymphocytic hypereosinophilic syndrome
M-HES: myeloproliferative hypereosinophilic syndrome
