# Supplementary material for: Tracking Public Interest in Rare Diseases and Eosinophilic Disorders in Germany: Web Search Analysis
Source: JMIR Infodemiology. 2025 May 26;5:e69040. doi: 10.2196/69040 (PMC12152774; doi:10.2196/69040)

**Table S1: Correlation matrix**

|  | Blood Eosinophilia | Churg strauss syndrome | EGPA | Eosinopenia | Eosinophils | Eosinophilic granulocytes | Eosinophilia | Eosinophilic fasciitis | Eosinophilic granulomatosis | fip1l1 | Hes | Mepolizumab | Nucala |
| --- | --- | --- | --- | --- | --- | --- | --- | --- | --- | --- | --- | --- | --- |
| Bad Bramst. | 298 | 13562 | 745 | 596 | 7377 | 3949 | 19523 | 2012 | 596 | 0 | 5738 | 7004 | 2832 |
| Berlin | 16 | 1184 | 52 | 59 | 2000 | 603 | 1965 | 139 | 33 | 6 | 680 | 210 | 226 |
| Bremen | 29 | 1043 | 48 | 51 | 1045 | 342 | 1513 | 183 | 71 | 2 | 587 | 207 | 218 |
| Cologne | 39 | 1623 | 106 | 115 | 2683 | 701 | 2772 | 211 | 79 | 6 | 1017 | 358 | 466 |
| Dortmund | 29 | 1183 | 41 | 129 | 2138 | 619 | 2386 | 194 | 73 | 8 | 863 | 228 | 359 |
| Dresden | 37 | 1750 | 94 | 119 | 2473 | 1545 | 2547 | 230 | 129 | 6 | 854 | 298 | 464 |
| Erfurt | 28 | 1892 | 57 | 108 | 1852 | 798 | 3066 | 296 | 120 | 0 | 690 | 439 | 826 |
| Frankfurt | 66 | 2628 | 97 | 149 | 4117 | 918 | 5029 | 300 | 100 | 4 | 2719 | 578 | 730 |
| Freiburg | 113 | 3313 | 409 | 191 | 3043 | 1200 | 4330 | 409 | 283 | 22 | 1413 | 896 | 700 |
| Giessen | 134 | 5779 | 188 | 497 | 7405 | 3387 | 10912 | 994 | 188 | 0 | 3198 | 1505 | 1707 |
| Hamburg | 31 | 1451 | 74 | 79 | 2356 | 556 | 2556 | 147 | 58 | 8 | 929 | 297 | 329 |
| Hanover | 76 | 2205 | 78 | 105 | 2300 | 953 | 2947 | 227 | 130 | 16 | 1085 | 413 | 435 |
| Heidelberg | 133 | 3279 | 251 | 230 | 4332 | 2379 | 6683 | 579 | 202 | 98 | 1598 | 795 | 970 |
| Jena | 74 | 3930 | 399 | 200 | 3520 | 1566 | 5537 | 525 | 420 | 42 | 1397 | 883 | 956 |
| Kassel | 113 | 3177 | 113 | 237 | 3820 | 2144 | 4992 | 458 | 175 | 0 | 1666 | 612 | 792 |
| Kiel | 107 | 2105 | 232 | 146 | 2195 | 1207 | 3239 | 369 | 176 | 17 | 1336 | 791 | 657 |
| Kirchheim/Teck | 75 | 4250 | 200 | 300 | 4225 | 2275 | 9075 | 675 | 150 | 0 | 1825 | 850 | 950 |
| Leipzig | 55 | 1945 | 127 | 115 | 2137 | 1008 | 2458 | 251 | 89 | 6 | 881 | 378 | 463 |
| Magdeburg | 76 | 2765 | 71 | 76 | 1467 | 1129 | 2285 | 213 | 116 | 4 | 676 | 365 | 413 |
| Mainz | 38 | 2119 | 65 | 216 | 2800 | 1243 | 3822 | 368 | 130 | 38 | 1124 | 605 | 562 |
| Mannheim | 54 | 1628 | 102 | 144 | 2242 | 923 | 3337 | 259 | 96 | 16 | 1073 | 444 | 498 |
| Munich | 39 | 2172 | 97 | 94 | 2928 | 1315 | 2794 | 195 | 78 | 23 | 985 | 450 | 470 |
| Nuremberg | 52 | 1590 | 48 | 88 | 1911 | 1012 | 2277 | 206 | 72 | 4 | 827 | 298 | 363 |
| Regensburg | 139 | 4723 | 372 | 201 | 3314 | 2540 | 5180 | 596 | 294 | 8 | 1665 | 999 | 883 |
| Rostock | 111 | 1947 | 161 | 86 | 2587 | 1004 | 4231 | 343 | 101 | 5 | 1044 | 469 | 489 |
| Stuttgart | 66 | 2150 | 92 | 168 | 3549 | 1332 | 4034 | 246 | 96 | 5 | 1433 | 373 | 468 |

**
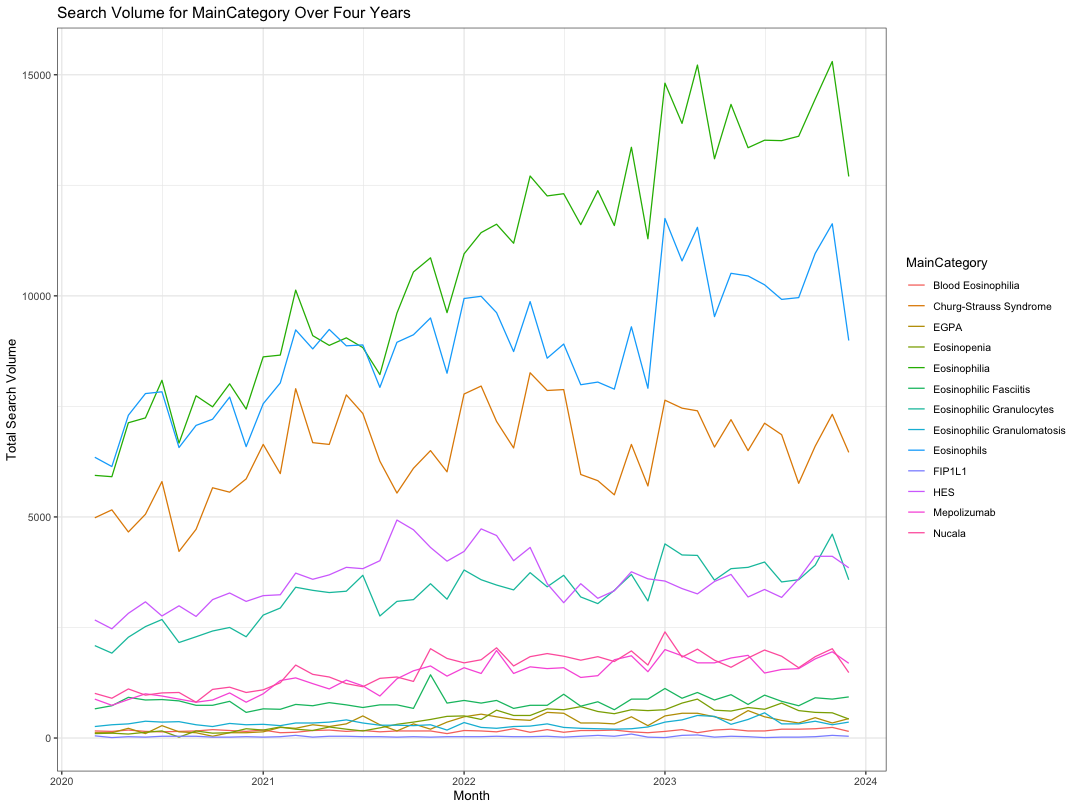
Figure S1: Total search volume per main category**

Figure S2: Trends in Cumulative Monthly Mentions Across Key Platforms (Facebook Public Posts, Forum Contributions, Online News, and X) with Peaks in January and November (Data Retrieved from Brandwatch[35])


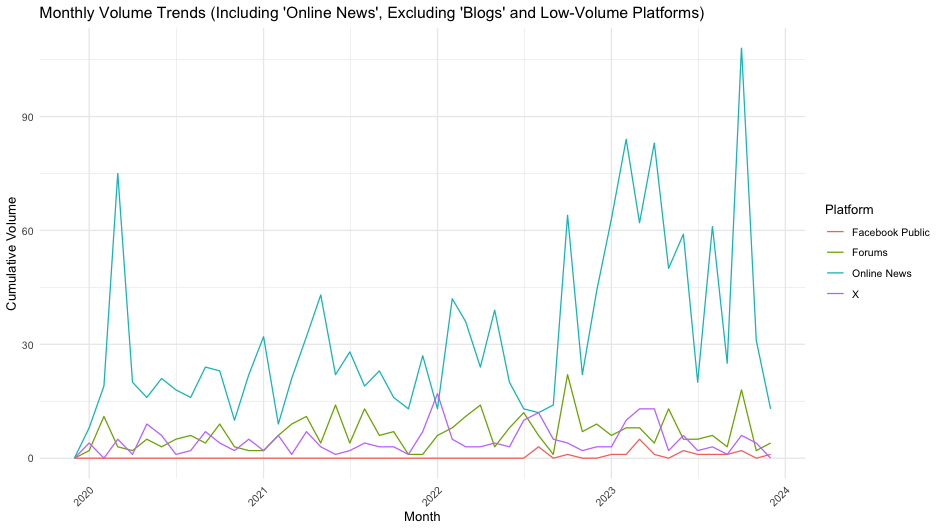


Figure S3: Search volume by main category over time by region: The strongest increase in search queries for "eosinophilia" is observed in several German states in 2023, particularly during the winter months.

**
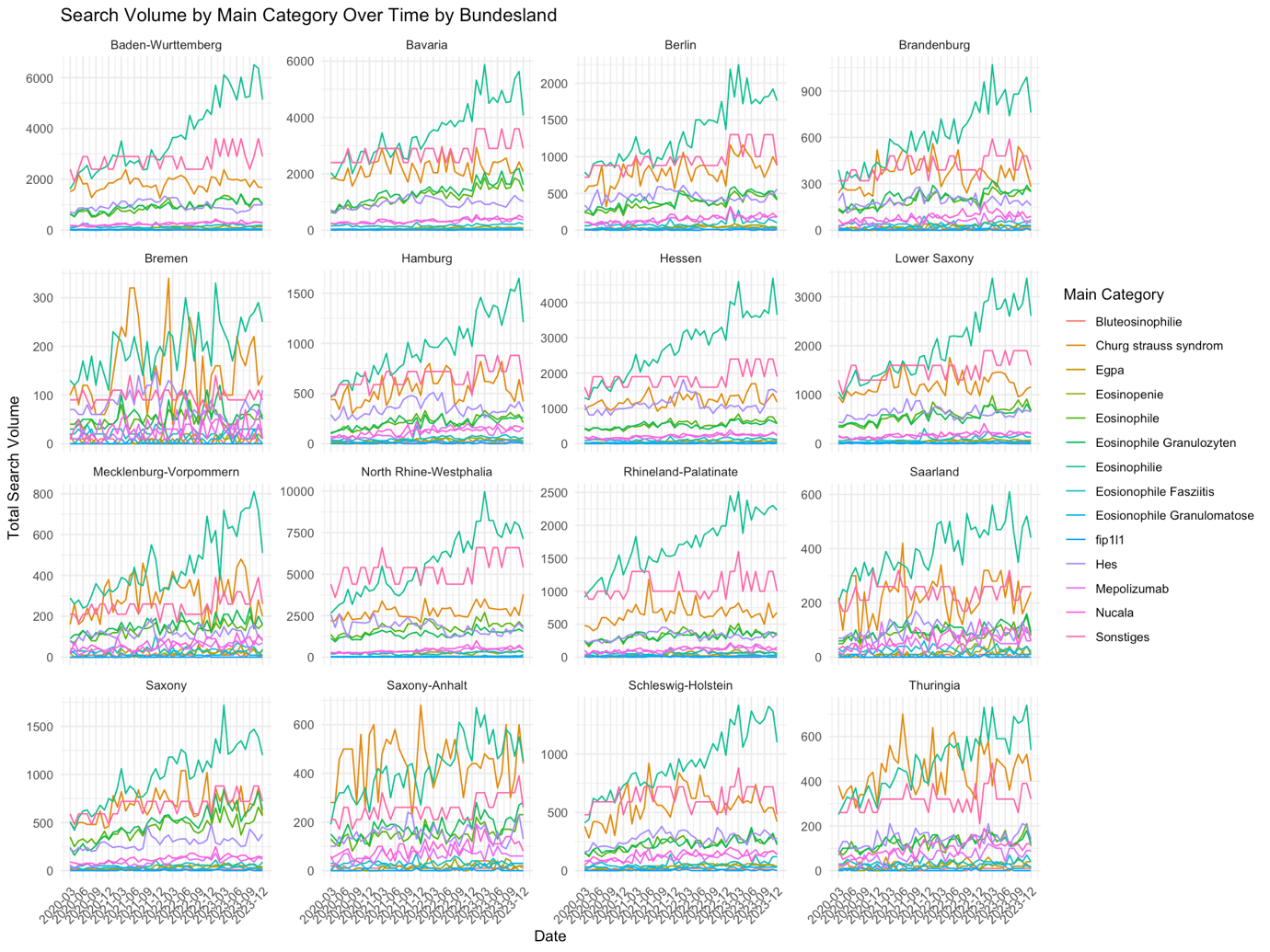
**

Figure S4: Search volume per 100,000 for top keywords in each city: The heatmap shows search volume per 100,000 inhabitants for the top 3 keywords ("Eosinophilia," "HES," and "granulocytes eosinophil") across various German cities. Bad Bramstedt stands out with high search volumes for "eosinophilia" and "HES." Regional differences are evident, with some cities showing much higher engagement than others, indicating varying levels of public interest in eosinophilic disorders.

**
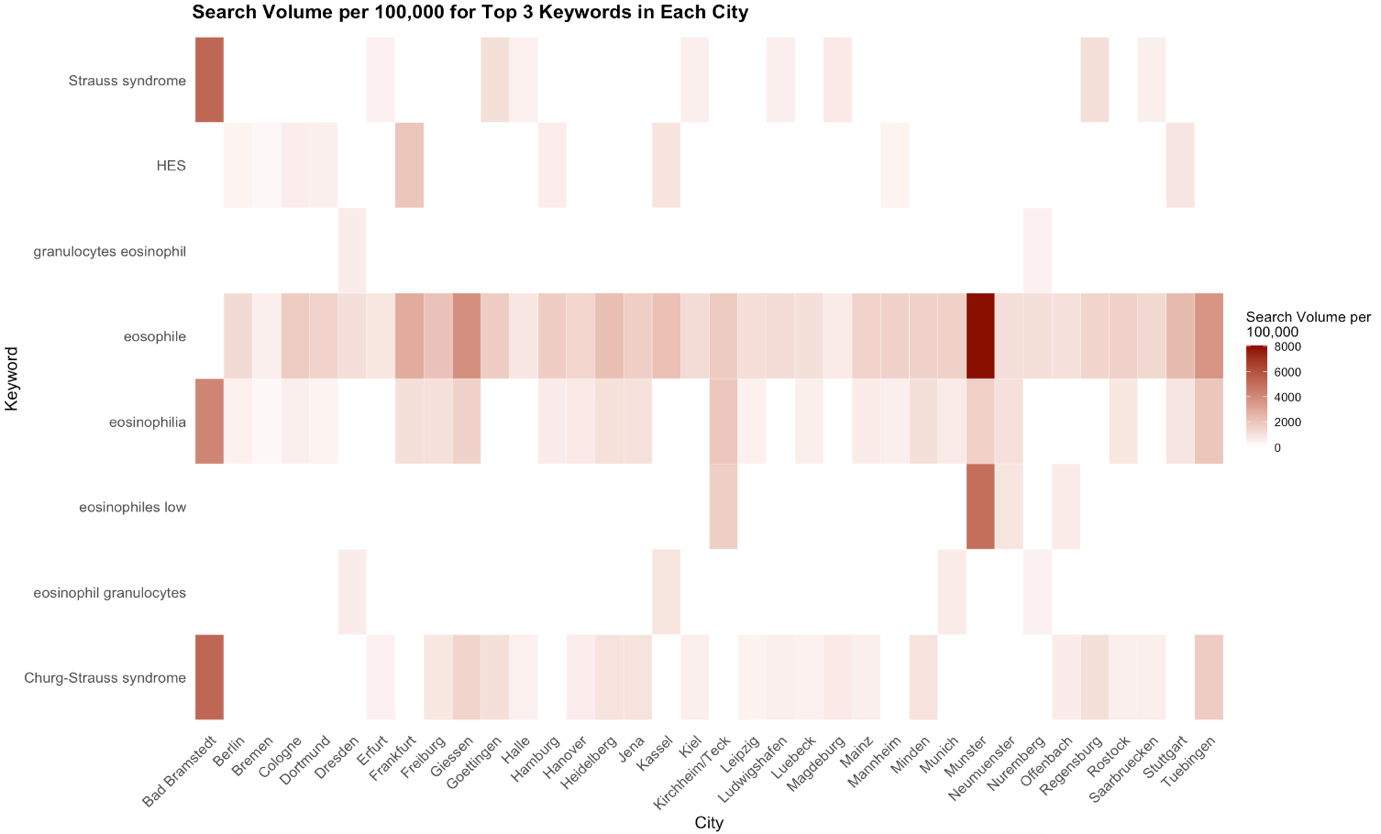
**

Figure S5: Decomposed Time Series Components: Seasonal, Trend, and Remainder


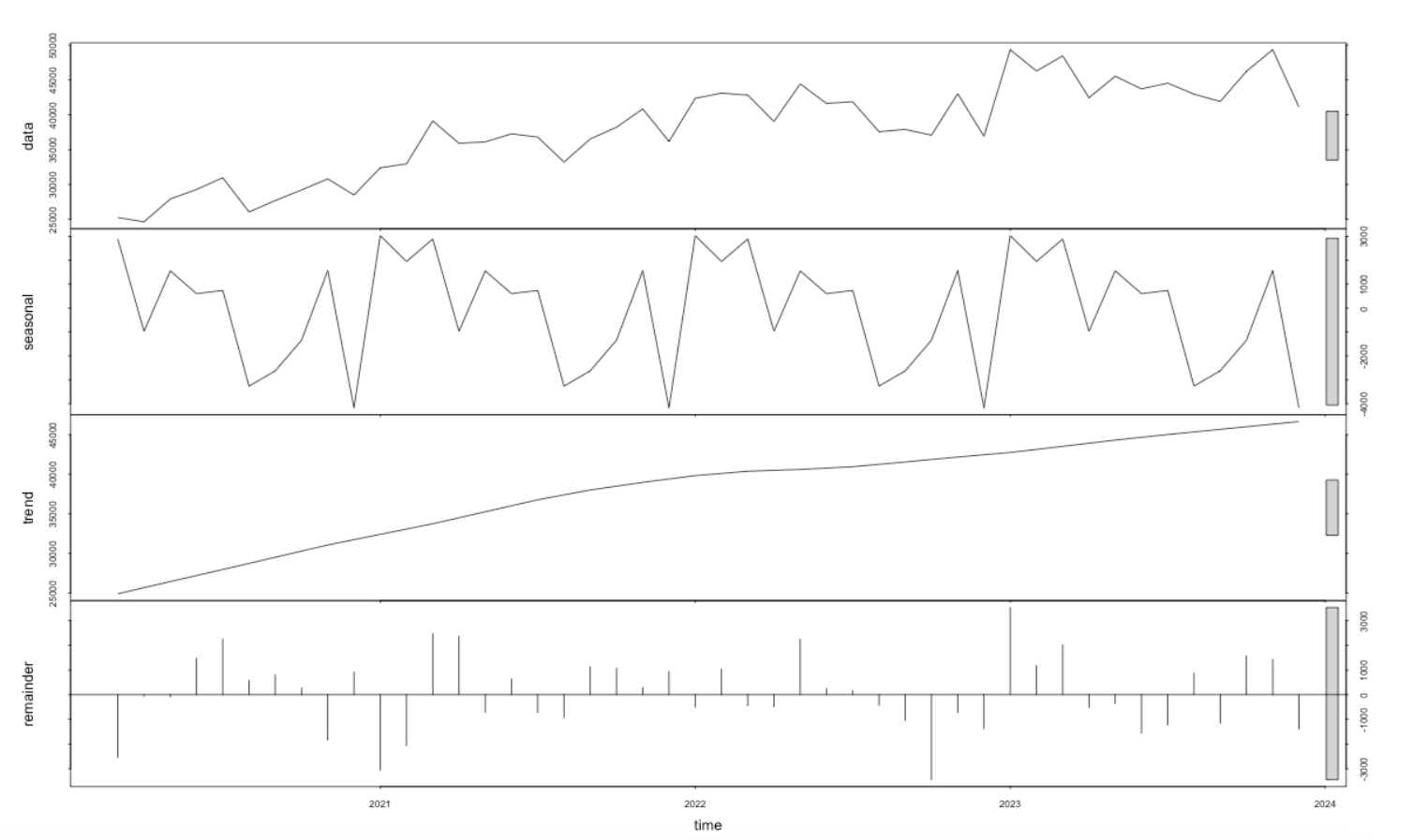


Figure S6: Decomposed Time Series Components: Seasonal, Trend, and Remainder


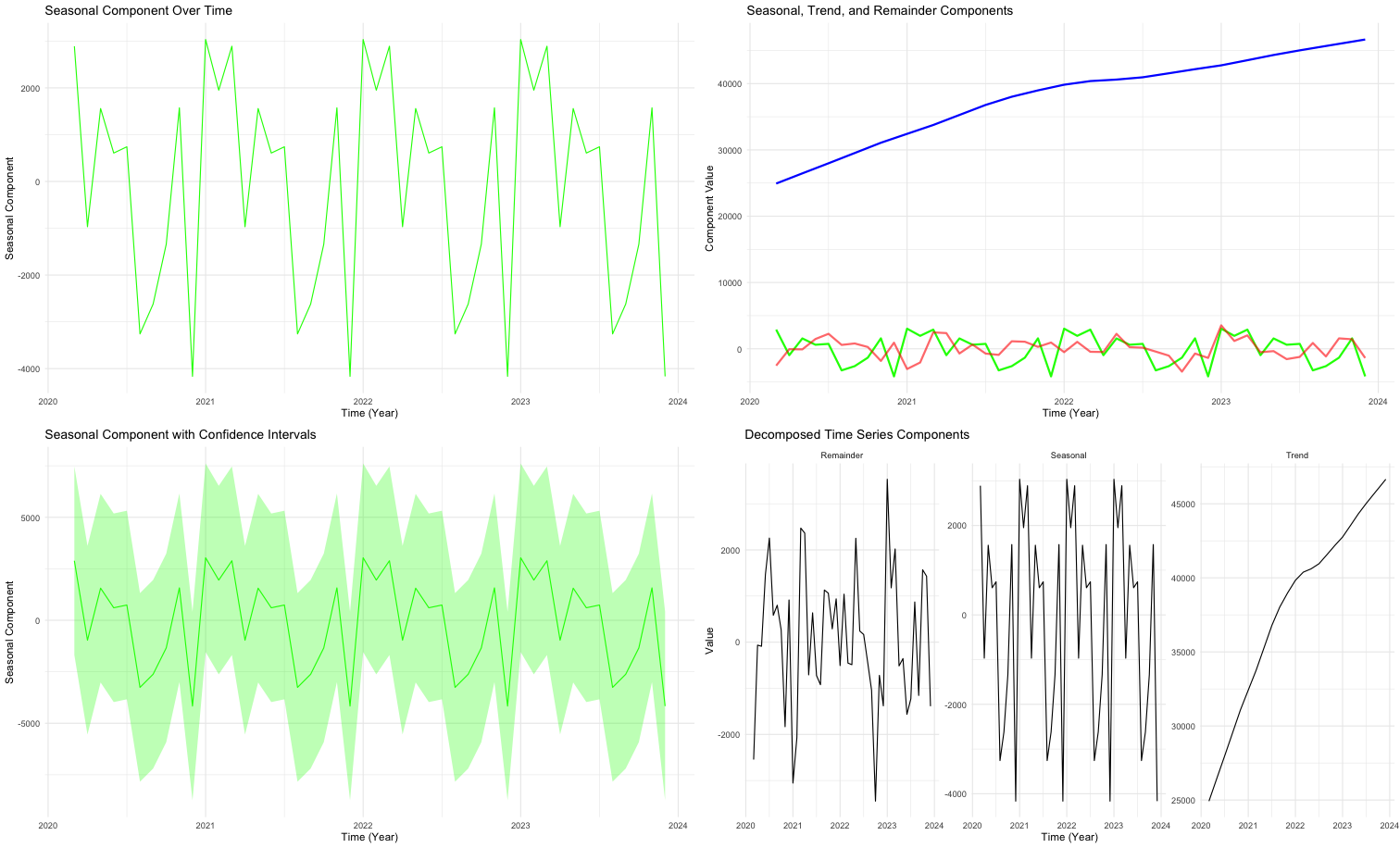

Supplement: Multimedia Appendix 1 [file infodemiology-v5-e69040-s001.docx]
